# Association between Bone Turnover, Micronutrient Intake, and Blood Lead Levels in Pre-and Postmenopausal Women, NHANES 1999–2002

**DOI:** 10.1289/ehp.1002158

**Published:** 2010-08-05

**Authors:** Leila W. Jackson, Barbara A. Cromer, Ashok Panneerselvamm

**Affiliations:** 1 Department of Epidemiology and Biostatistics, Case Western Reserve University School of Medicine, Cleveland, Ohio, USA; 2 Department of Pediatrics, MetroHealth Medical Center, Case Western Reserve University, Cleveland, Ohio, USA

**Keywords:** bone resorption, calcium, iron, lead, osteogenesis, premenopause, postmenopause, vitamin D

## Abstract

**Background:**

Blood lead levels (BLLs) have been shown to increase during periods of high bone turnover such as pregnancy and menopause.

**Objectives:**

We examined the associations between bone turnover and micronutrient intake with BLLs in women 20–85 years of age (*n* = 2,671) participating in the National Health and Nutrition Examination Survey, 1999–2002.

**Methods:**

Serum bone-specific alkaline phosphatase (BAP) and urinary cross-linked N-telopeptides (NTx) were measured as markers of bone formation and resorption, respectively. Lead was quantified in whole blood. The association between tertiles of BAP and NTx, and BLLs was examined using linear regression with natural log_-_transformed BLLs as the dependent variable and interpreted as the percent difference in geometric mean BLLs.

**Results:**

In adjusted analyses, mean BLLs among postmenopausal women in the upper tertiles of NTx and BAP were 34% [95% confidence interval (CI), 23%–45%] and 30% (95% CI, 17%–43%) higher than BLLs among women in the lowest tertiles of NTx and BAP, respectively. These associations were weaker, but remained statistically significant, among premenopausal women (NTx: 10%; 95% CI, 0.60%–19%; BAP: 14%; 95% CI, 6%–22%). Within tertiles of NTx and BAP, calcium intake above the Dietary Reference Intake (DRI), compared with below the DRI, was associated with lower mean BLLs among postmenopausal women but not premenopausal women, although most of the associations were not statistically significant. We observed similar associations for vitamin D supplement use.

**Conclusions:**

Bone resorption and bone formation were associated with a significant increase in BLLs among pre-and postmenopausal women.

Despite successful efforts to reduce sources of lead exposure over the past several decades, lead remains ubiquitous in the environment, providing a source of exposure to humans. In addition, lead bioaccumulates in bone, where it has a half-life of approximately 12–27 years, providing another source of exposure when it is mobilized (Bergdahl et al. 1998; Gerhardsson et al. 1993). Previous research has shown that blood lead levels (BLLs) increase during periods of high bone turnover such as pregnancy (Gulson et al. 1998, 1999; Hertz-Picciotto et al. 2000; Manton et al. 2003; Rothenberg et al. 1994), lactation (Gulson et al. 1998, 1999; Manton et al. 2003; Téllez-Rojo et al. 2002), menopause (Garrido-Latorre et al. 2003; Gulson et al. 2002; Korrick et al. 2002; Silbergeld et al. 1988; Symanski and Hertz-Picciotto 1995), and severe weight loss (Riedt et al. 2009), with levels modified by calcium intake (Ettinger et al. 2009; Hernandez-Avila et al. 2003; Hertz-Picciotto et al. 2000; Janakiraman et al. 2003). This increase in BLLs may put an individual at an increased risk for a variety of adverse health outcomes, because there is currently no known safe level of lead in blood.

To date, few studies have examined the association between markers of bone resorption and formation, and BLLs. Among pregnant women (*n* = 193) with high bone lead levels, Téllez-Rojo et al. (2004) found that plasma and whole BLLs increased significantly with increased bone resorption as measured by cross-linked N-telopeptides (NTx). Within a population of Japanese women (*n* = 1,225), investigators observed significantly higher BLLs in relation to bone formation levels above the median among perimenopausal women regardless of bone resorption levels, but not among pre-or postmenopausal women (Machida et al. 2009).

Because bone is continuously being remodeled, resulting in a constant, low-level exchange of lead between the blood and bone compartments (Rabinowitz et al. 1976), periods of low to moderate bone turnover may also be of importance among women with high bone lead levels (Silbergeld et al. 1993); however, little research has been done in this area. For this reason, as well as ongoing concerns associated with the adverse health effects of increased BLLs among adults (Berg 2009), we examined associations among bone formation and resorption, micronutrient intake, and BLLs in women 20–85 years of age participating in the National Health and Nutrition Examination Survey (NHANES) 1999–2002.

## Materials and Methods

### Data source

NHANES is a cross-sectional, national survey that assesses the health and nutrition of children and adults in the United States. In 1999, the survey became a continuous, annual survey, with approximately 5,000 individuals interviewed per year. The study population is a stratified, multistage probability sample of the civilian, noninstitutionalized U.S. population. From 1999 to 2002, the study was oversampled for low-income persons, adolescents, persons > 60 years of age, African Americans, and Mexican Americans. NHANES includes both a household interview and mobile examination component. Detailed information regarding the NHANES survey can be found from the study Web site [Centers for Disease Control and Prevention (CDC) 2010]. This secondary data analysis was approved by the University Hospitals Case Medical Center Institutional Review Board under the exempt research category.

### Study population

Women 20–85 years of age who participated in the mobile examination component and who completed the reproductive health questionnaire were eligible for analyses (*n* = 4,582). The study population was further limited to women with complete data on BLLs and markers of bone turnover (*n* = 4,255). Women were excluded if they fell into one or more of the following categories: menopausal status could not be determined (*n* = 80); pregnant (*n* = 470); lactating (*n* = 91); reported current thyroid disease (*n* = 140) or ever being diagnosed with diabetes (*n* = 371), kidney disease (*n* = 102), liver disease (*n* = 50), or cancer other than nonmelanoma skin cancer (*n* = 293); or had data missing on important confounders (*n* = 343). The final study population included 2,671 women.

Menopausal status was determined by responses on the reproductive health questionnaire. Women reporting regular cycles and/or their last menstrual period within the preceding 60 days were considered premenopausal. Women reporting their last menstrual period in the preceding 3–11 months were considered late perimenopausal unless they indicated “medical conditions/treatments” as a reason for their irregular menstrual cycles. Women who reported not having a menstrual period in the preceding 12 months unless due to “medical conditions/treatments” or who reported having a hysterectomy and/or bilateral oophorectomy were considered postmenopausal. If women reported irregular cycles due to “medical conditions/treatments” and did not have a menstrual period in the preceding 60 days, they were classified as “unable to determine.” Follicle-stimulating hormone levels were used to help classify menopausal status for approximately 10% of women 35–60 years of age whose menstrual cycle data were missing or inconsistent.

### Measurement of bone turnover, lead, and micronutrients

NHANES measured bone-specific alkaline phosphatase (BAP), a marker of bone formation, in serum by either the Hybritech Tandem-MP Ostase ImmunoEnzyme assay (NHANES 1999–2001) or Beckman Access Ostase assay (NHANES 2002). NTx, a marker of bone resorption, was measured in urine by competitive-inhibition enzyme-linked solid-phase immunosorbent assay (Osteomark; NHANES 1999–2001) or Vitros Eci competitive assay (NHANES 2002). All NTx values were creatinine adjusted. Given the change in laboratory methods for both BAP and NTx during the 2001–2002 study period, NHANES adjusted the measured values to account for the observed variability across methods (CDC 2008). An additional adjustment was made to BAP levels to trend the data over the full survey period, 1999–2002, using regression methods recommended by NHANES (CDC 2008). For the measurement of lead, blood was collected in EDTA-anticoagulant Vacutainers that were prescreened for background metal contamination. Lead was quantified in whole blood with a SIMAA 6000 simultaneous multielement atomic absorption spectrometer (Perkin-Elmer, Waltham, MA) with Zeeman background correction, with a minimum detection level of 0.6 μg/dL and coefficients of variation ranging from 3.1% to 7.0%.

Data on use of calcium, iron, and vitamin D supplements were based on self-reported use in the past month. Each supplement reported by the participant was recorded, as well as the frequency of use and quantity taken. Detailed data on supplement ingredients were abstracted by NHANES staff, allowing for quantification of micronutrient supplement intake. Dietary nutrient intake of calcium and iron was quantified using a 24-hr dietary recall. Supplement and dietary intake were summed to estimate the total milligrams of calcium and iron intake per day, and dichotomized as less than or greater than/equal to the Dietary Reference Intake (DRI) for calcium (women 19–50 years of age, 1,000 mg/day; > 50 years of age, 1,200 mg/day) and iron (women 19–50 years of age, 18 mg/day; > 50 years of age, 8 mg/day) (Standing Committee on the Scientific Evaluation of Dietary Reference Intakes 1997, 2000). Because dietary data were not available for vitamin D and very few women had intakes above the DRI based on supplement use, vitamin D was analyzed as vitamin D supplement use in the past month (yes/no).

### Measurement of confounders

Information on menarche, parity, sex hormone use, years since menopause (age at interview minus age at last menstrual period), and surgical menopause was obtained from the reproductive health questionnaire. Surgical menopause was categorized as no history of hysterectomy or bilateral oophorectomy, history of hysterectomy without bilateral oophorectomy, or history of bilateral oophorectomy with or without hysterectomy. Women who reported currently using hormonal contraception or hormone replacement therapy were categorized as currently using sex hormones.

A single variable indicating level of physical exercise was created based on self-reported moderate and vigorous activity over the preceding 30 days (no activity, moderate, and vigorous). A woman was considered to have participated in muscle-strengthening activities in the preceding 30 days if she reported doing any “physical activities specifically designed to strengthen your muscles such as lifting weights, push-ups, or sit-ups” during that time period. Osteoporosis and family history of osteoporosis were based on self-report by the woman of herself or her biological relatives (living and deceased), respectively, ever being told by a health professional that they had osteoporosis or brittle bones.

Data on other factors of interest such as demographics, body mass index (BMI), alcohol and cigarette use, and housing characteristics were obtained from their respective questionnaires. Poverty income ratio, as calculated by NHANES, was used as a measure of socioeconomic status and is the ratio of income to the family’s appropriate poverty threshold as determined by family size and composition. Values < 1.00 represent a family living below the official poverty threshold, whereas values ≥ 1.00 indicate income at or above the poverty level. Poverty income ratio was dichotomized for analyses (< 2.0 and ≥ 2.00). BMI was categorized according to World Health Organization criteria (< 25.0, 25.0–29.9, and ≥ 30 kg/m^2^) (World Health Organization 1995).

### Statistical analysis

Geometric mean BLLs were compared across categories of demographic, lifestyle, and reproductive health factors using the Kruskal–Wallis test, because BLLs were not normally distributed. The associations between tertiles of NTx and BAP, and BLLs were examined using linear regression with natural log-transformed BLLs as the dependent variable. Results were back-transformed and interpreted as the percent difference in geometric mean BLLs compared with levels in the lowest tertile of exposure. BAP and NTx were modeled separately because they were highly correlated (*r* = 0.37; *p* < 0.001). Factors shown in the literature to be associated with BLLs, NTx, or BAP or that changed the association between markers of bone turnover and BLLs by > 10% were examined as potential confounders; all variables were entered as categorized in Table 1 except age, which was entered as a continuous variable. Only those variables that confounded the association between exposure and outcome were retained in the final models. Further analyses were stratified by menopausal status; late perimenopausal women were not examined separately given the small number of women. In subanalyses, we examined the association between NTx and BLLs within tertiles of BAP.

To examine associations between supplement use or micronutrient intake and percent difference in mean BLLs, any supplement use in the past month (yes/no), total daily calcium and iron intake (dichotomized at DRI), and vitamin D supplement use in the past month (yes/no) were modeled independently and in a single model with adjustment for potential confounders, including NTx and BAP. We further examined potential interactions between each micronutrient, and BAP and NTx in adjusted analyses.

The 4-year mobile-examination-component sample weights were used for regression analyses taking into account the complex sampling design of NHANES and nonresponse of eligible participants. Sampling errors were estimated using the Taylor series linearized method. Study results were considered statistically significant if the *p*-value was < 0.05; however, some results may be significant by chance. All analyses were completed in STATA 9.0 (StataCorp LP, College Station, TX).

## Results

Among eligible women, 1,359 were premenopausal, 58 late perimenopausal, and 1,254 postmenopausal (median age since last menstrual period = 17 years; interquartile range = 8–27 years). The geometric mean BLL for the overall population was 1.44 μg/dL [95% confidence interval (CI), 1.41–1.48 μg/dL], with peri-and postmenopausal women having higher BLLs compared with premenopausal women (Table 1). White non-Hispanic women had lower mean BLLs than did black non-Hispanic women. Women who consumed one or more drinks per week or were former or current smokers had higher BLLs than did women who consumed less than one drink per week or were never-smokers, respectively. Exercise or muscle-strengthening activities in the preceding 30 days, current use of sex hormones, and calcium intake above the DRI were associated with lower BLLs, whereas iron intake above the DRI was associated with higher BLLs. Women who reported being diagnosed with osteoporosis had higher BLLs than did those who did not have osteoporosis, whereas those with a family history of osteoporosis had lower BLLs than did those without a family history. BLLs were higher among women living in homes built before 1960 (vs. 1960 or later), having their NHANES examination between 1 May and 31 October (vs. between 1 November and 30 April), and not using a water treatment device in their home (vs. using a device; data not shown).

Geometric mean BLLs increased by tertiles of both BAP and NTx (*p*-value for trend < 0.001) in the overall study population (Table 1). Women in the second and third tertiles of NTx and BAP had higher mean BLLs than did women in the lowest tertile of each exposure (Table 2). After adjusting for age, menopausal status, race/ethnicity, current hormone use, surgical menopause, smoking status, and BMI, mean BLLs were significantly higher among women in the second and third tertiles of NTx relative to the lowest tertile (5% higher; 95% CI, 0.13–11.06%; 18% higher; 95% CI, 11.63–24.78%, respectively). Adjusted mean BLLs were also significantly higher among women in the second (7%; 95% CI, 1.09–14.28%) and third (20%; 95% CI, 13.68–27.44%) tertiles of BAP compared with the first, respectively. These results did not change in sensitivity analyses in which *a*) we included in analyses women originally excluded for exclusionary diagnoses, and *b*) we excluded from analyses women with osteoporosis or family history of osteoporosis.

NTx and BAP were also associated with higher mean BLLs when we examined premenopausal and postmenopausal women separately (Table 3). Among premenopausal women, adjusted mean BLLs were 10% (95% CI, 0.60–19.21%) and 14% (95% CI, 6.49–22.08%) higher for women in the highest versus lowest tertile of NTx and BAP, respectively. Among postmenopausal women, adjusted mean BLLs were 15% (95% CI, 5.18–26.75%) and 34% (95% CI, 23.31–45.39%) higher for women in the second and third tertiles of NTx, and 13% (95% CI, 1.12–25.99%) and 30% (95% CI, 17.93–43.36%) higher for women in the second and third tertiles of BAP, relative to women in the first tertile of each exposure. These associations did not change after adjusting for years since menopause, although BLLs decreased by approximately 0.8% for each additional year since menopause (NTx: −0.73%; 95% CI, −1.29% to −0.17%; BAP: −0.80; 95% CI, −1.36% to −0.24%).

**Figure 1:**
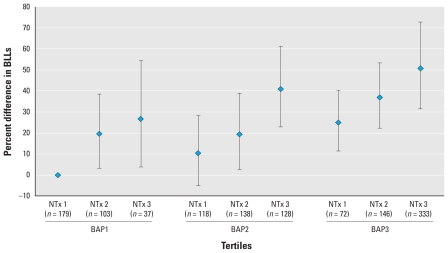
Percent difference (95% CI) in geometric mean BLLs by tertiles of bone formation (serum BAP) and resorption (creatinine-adjusted urinary NTx), relative to the lowest tertiles of BAP and NTx and adjusted for age, race/ethnicity, current hormone use, smoking status, BMI, surgical menopause, supplement use, and alcohol consumption: postmenopausal women, NHANES 1999–2002 (*n* = 1,254).

We explored the association between NTx and BLLs within tertiles of BAP for both pre-and postmenopausal women. Within each tertile of BAP, premenopausal women with higher NTx levels had higher mean BLLs than did women with lower NTx levels. The adjusted mean BLL in postmenopausal women in the highest tertiles of both NTx and BAP was 51% higher (95% CI, 31.45–72.81%) than women in the lowest tertiles of both exposures (Figure 1). We observed similar patterns for premenopausal women: Mean adjusted BLL in women in the highest tertiles of both NTx and BAP was 19% (95% CI, 5.66–34.78%) higher than in women in the lowest tertiles of both BAP and NTx [Supplemental Material, Figure 1 (doi:10.1289/ehp.1002158)]. Despite these findings, we found no statistically significant multiplicative interaction between BAP and NTx for either pre-or postmenopausal women.

### Micronutrient intake

In postmenopausal women, use of any supplements and use of vitamin D supplements in the past month were both associated with significantly lower adjusted mean BLLs, whereas we found a nonsignificant difference with calcium intake above and below the DRI (Table 4). These associations did not change substantially after adjusting for iron and for calcium or vitamin D intake. Iron intake was associated with slightly lower mean BLLs among postmenopausal women (Table 4); however, this association weakened after adding calcium and vitamin D to the models. Any supplement use was associated with lower mean BLLs among premenopausal women (NTx: −9.54% ; 95% CI, −16.68% to −1.79%; BAP: −9.15%; 95% CI, −16.50% to −1.16%); however, we found no association between specific micronutrients and BLLs in this group (data not shown).

**Figure 2:**
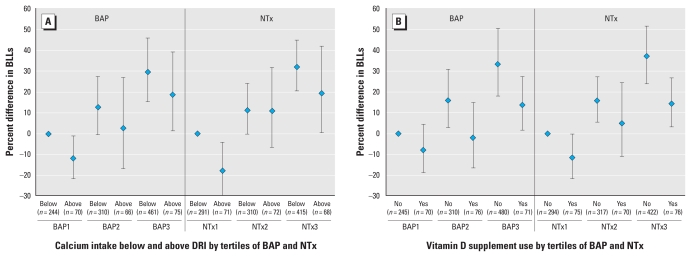
Percent difference (95% CI) in geometric mean BLLs by type of micronutrient and tertiles of bone formation (serum BAP) and resorption (creatinine-adjusted urinary NTx), relative to no intake or use in the lowest tertile of BAP or NTx and adjusted for age, race/ethnicity, current hormone use, smoking status, BMI, surgical menopause, supplement use, and alcohol consumption: postmenopausal women, NHANES 1999–2002. (*A*) Calcium intake below and above the DRI (*n* = 1,226). (*B*) Vitamin D supplement use in the past month (*n* = 1,254).

Neither micronutrient intake nor supplement use appeared to modify associations between BAP or NTx and BLLs. Among postmenopausal women, any supplement use (vs. no use) [Supplemental Material, Figure 2 (doi:10.1289/ehp.1002158)], calcium use at or above the DRI (vs. < DRI) (Figure 2A), and vitamin D supplement use (vs. no use) (Figure 2B) were associated with lower mean BLLs within tertiles of BAP and NTx. Within the lowest tertile of BAP and NTx, respectively, postmenopausal women with calcium intake above the DRI had 12% (95% CI, −21.67% to −0.97%) and 18% (95% CI, −29.63% to −4.04%) lower mean BLLs compared with those with intake below the DRI after adjusting for potential confounders (Figure 2A). Vitamin D supplement use did not significantly alter mean BLLs; however, we found some evidence that it was associated with lower mean BLLs, as observed for calcium (Figure 2B). Iron intake among postmenopausal women did not greatly alter BLLs [Supplemental Material, Figure 3 (doi:10.1289/ehp.1002158)]. Among premenopausal women, any supplement use in the past month was associated with lower mean BLLs across most tertiles of NTx and BAP [Supplemental Material, Figure 4 (doi:10.1289/ehp.1002158)]; however, we found no clear association for specific micronutrients.

## Discussion

In this representative sample of the U.S. noninstitutionalized female population, we found that both bone formation (serum BAP) and bone resorption (creatinine-adjusted urinary NTx) were significantly associated with higher mean BLLs among pre-and postmenopausal women. Furthermore, calcium intake and vitamin D supplement use among postmenopausal women were associated with lower mean BLLs across most tertiles of NTx and BAP, although the effect estimates were imprecise.

Although many sources of lead exposure have been reduced or eliminated over the past three decades, lifetime exposure to these sources remains a threat to human health because of the bioaccumulation of lead in bone. Approximately 92% of lead that enters the body will be excreted through urine and feces (International Programme on Chemical Safety 1995); however, 90% of the remaining lead is taken up by the bones and teeth, where it has a half-life of 12–27 years (Bergdahl et al. 1998; Gerhardsson et al. 1993). Higher bone lead levels have been associated with increasing age, black race, increasing pack-years of smoking, increasing alcohol intake, lower parity, never breast-feeding, use of lead-glazed pottery, and occupational lead exposure (Brown et al. 2000; Korrick et al. 2002; Lin et al. 2004; Popovic et al. 2005). Those with higher bone lead stores may be at an increased risk for higher BLLs when lead is mobilized from bone tissue during regular bone turnover and periods of high bone turnover, as observed in this study.

Bone is continuously being remodeled; however, periods of high turnover may be of particular concern (Roberts and Silbergeld 1995). Within our analyses of postmenopausal women, those in the upper tertile of both BAP and NTx had an estimated mean BLL that was 51% higher than the mean BLL of women in the lowest tertiles of BAP and NTx. Furthermore, we observed significant positive associations with high (vs. low) BAP among women with low NTx, and with high (vs. low) NTx among women with low BAP (Figure 1), consistent with potential independent effects of bone formation and bone resorption on BLLs, respectively. The independent association of BAP with BLL was unexpected but may reflect the true physiology of bone turnover in which new bone formation closely follows bone resorption in any given segment of bone; however, it may be difficult to truly separate these effects statistically given their strong correlation. Among a population of perimenopausal women, Machida et al. (2009) observed a similar independent effect of bone formation (BAP) on BLLs, regardless of the level of bone resorption (NTx). Alternatively, the association we observed may be due to residual confounding related to the assay or the use of tertiles, or may be due to chance. Potula et al. (2005) observed a significant increase in BLLs related to menopausal stage, similar to our observations; however, in their adjusted analyses (*n* = 73) neither bone formation nor bone resorption was significantly associated with BLLs in pre-or postmenopausal women.

Our results provide further support for studies that observed higher BLLs among peri-and postmenopausal women compared with premenopausal women but did not measure markers of bone turnover (Garrido-Latorre et al. 2003; Gulson et al. 2002; Korrick et al. 2002; Silbergeld et al. 1988; Symanski and Hertz-Picciotto 1995). Several of these studies reported significant associations between bone lead levels and BLLs while adjusting for other factors (Berkowitz et al. 2004; Garrido-Latorre et al. 2003; Korrick et al. 2002). As shown by Téllez-Rojo et al. (2004) in a cohort of pregnant women, both the bioavailability of lead in bone and the level of bone resorption are important predictors of BLLs. Although we did not have bone lead measurements, the stronger association observed among postmenopausal women compared with premenopausal women within the same tertiles of bone formation and resorption may be explained by potentially higher bone lead stores in postmenopausal women due to higher lifetime exposures and a longer duration of exposure, and therefore increased bioavailability of lead in bone.

Lead exposure remains a concern given the growing number of adverse health effects observed in both children and adults at levels well below the current levels of concern set by the CDC (1991; children and pregnant women, 10 μg/dL; adults, 25 μg/dL) and the Occupational Safety and Health Administration (1978; 40 μg/dL), with no known safe level of lead in blood currently identified. Higher BLLs in adults have been associated with adverse pregnancy and reproductive outcomes (Bellinger 2005; Chang et al. 2006; Popovic et al. 2005; Tang and Zhu 2003), chronic kidney and cardiovascular disease (Navas-Acien et al. 2007, 2009), impaired cognitive function (Weuve et al. 2009), osteoporosis (Campbell and Auinger 2007), fractures (Khalil et al. 2008), menopausal age (Popovic et al. 2005), and overall and cardiovascular mortality (Weisskopf et al. 2009); therefore, it is important to identify factors that may modify the association between bone formation and resorption and BLLs. To this end, we examined associations between calcium, iron, and vitamin D intake and BLLs, given their importance in lead absorption and/or bone turnover (Avendano-Badillo et al. 2009; Ettinger et al. 2009; Hernandez-Avila et al. 2003; Hertz-Picciotto et al. 2000; Janakiraman et al. 2003). Calcium and vitamin D intake were associated with lower mean BLLs across most tertiles of NTx and BAP among postmenopausal women, but not among premenopausal women. A similar association between calcium and BLLs has been observed among pregnant and lactating women (Ettinger et al. 2009; Hernandez-Avila et al. 2003; Hertz-Picciotto et al. 2000), consistent with the increased bone turnover experienced by these women. In contrast, Potula et al. (2005) observed a significant positive association between serum ionized calcium levels and BLLs among postmenopausal women (*n* = 47), but a nonsignificant, inverse relationship among premenopausal women (*n* = 26) in adjusted analyses; they observed no association between serum vitamin D levels and BLLs.

Given the cross-sectional nature of this analysis, it is not possible to determine whether bone formation and resorption are causally associated with increased BLLs. Furthermore, it is unclear where micronutrient intake may fall in the causal pathway because some women may increase their intake to prevent bone resorption or osteoporosis, whereas others may increase intake as treatment for these conditions. For this reason, we excluded women with medical conditions that may alter bone resorption or formation. In sensitivity analyses, the addition of these women to the analyses, as well as the exclusion of women with osteoporosis or family history of osteoporosis from the analyses, did not change the associations between NTx and BAP, and BLLs.

Using data on both supplement use and dietary intake, we were able to estimate the daily intake of calcium and iron in relation to DRIs. We estimated vitamin D intake using self-reported vitamin D supplement use only, which may have resulted in measurement error for this variable because the primary sources of vitamin D are through diet and sunlight exposure; therefore, these results should be interpreted cautiously. Given that micronutrient exposures do not occur singularly, but rather as mixtures, in our primary analyses we used “any supplement use in the past month,” which was a stronger predictor of BLLs than any one micronutrient alone. In secondary analyses, we also generated models including all three micronutrients and did not find major changes in the point estimates for BAP or NTx.

NTx levels in our study were similar to those observed in the Study of Women’s Health Across the Nation (Sowers et al. 2003) but lower than those observed by Machida et al. (2009), as were BAP levels. These differences may be attributable to differences in study populations and eligibility criteria, timing of urine collection, or both. Machida et al. (2009) appropriately obtained samples from a second-morning urinary void, whereas collection of samples was untimed for NHANES. Despite the lower levels of NTx and BAP observed in our analyses compared with those of Machida et al. (2009), we still observed a significant association between these markers and BLLs.

Finally, based on the NHANES data, we could not delineate whether lead as measured in blood was primarily from bone stores or from new sources of environmental or occupational lead exposure. We did examine age of homes as an indicator of lead paint in the home, season of examination as a marker of increased environmental exposure during the summer months, and use of water treatment devices as a means of removing lead from water in the home. Age of home was missing for a large proportion of individuals (21%), and although living in homes built before 1960 was associated with significantly higher BLLs, it did not change the estimates for BAP or NTx in adjusted analyses. Season (1 May to 31 October vs. 1 November to 30 April) was associated with significantly higher BLLs, and use of water treatment devices with significantly lower BLLs, but neither confounded the associations between NTx and BAP, and BLLs.

## Conclusions

Within this study of “healthy” pre-, peri-, and postmenopausal women participating in NHANES 1999–2002, we observed significantly higher mean BLLs with increasing levels of bone formation (serum BAP) and resorption (creatinine-adjusted urinary NTx). We observed 20% and 51% increases in BLLs among pre-and postmenopausal women, respectively, who were in the highest tertiles of bone formation and resorption compared with those in the lowest tertiles. This suggests that women with higher levels of bone formation and resorption may be at increased risk for lead-associated morbidity and mortality. Among postmenopausal women, but not premenopausal women, calcium and vitamin D intake were associated with lower mean BLLs. Further elucidation of associations among bone lead levels, bone turnover, micronutrient intake, and BLLs is needed in prospective studies. Health care providers should continue to emphasize the importance of adequate calcium and vitamin D intake among women to prevent osteoporosis and other adverse health conditions, as well as to reduce the potential impact of bone turnover on BLLs.

## Figures and Tables

**Table 1:** Geometric mean BLLs (unweighted) by population characteristics of women 20–85 years of age participating in NHANES 1999–2002 (*n* = 2,671).

Variable	*n*	Geometric mean (μg/dL)	95% CI^a^
NTx (nM bone collagen equivalent/mM creatinine)			
0–27.39	891	1.29	1.25–1.34
27.40–40.76	890	1.36	1.31–1.42
40.77–934.58	890	1.71	1.63–1.78

BAP (μg/L)			
0–10.30	901	1.23	1.19–1.28
10.31–14.40	886	1.39	1.34–1.45
14.41–153.00	884	1.76	1.68–1.83

Age (years)			
20–34	678	1.00	0.96–1.05
35–59	1,190	1.45	1.40–1.50
≥ 60	803	1.95	1.88–2.03

Menopausal status			
Premenopausal	1,359	1.14	1.10–1.18
Late perimenopausal	58	1.69	1.45–1.98
Postmenopausal	1,254	1.85	1.79–1.91

Race/ethnicity			
Non-Hispanic white	1,300	1.39	1.34–1.43
Non-Hispanic black	512	1.56	1.47–1.65
Other	859	1.47	1.40–1.53

Poverty income ratio			
< 2.00	1,010	1.54	1.47–1.60
≥ 2.00	1,400	1.33	1.29–1.37
Missing	261	1.77	1.64–1.90

BMI (kg/m^2^)			
< 25.0	941	1.44	1.38–1.50
25.0–29.9	817	1.53	1.46–1.59
≥ 30.0	913	1.38	1.32–1.44

Osteoporosis			
No	2,495	1.43	1.39–1.46
Yes	176	1.67	1.54–1.82

Family history of osteoporosis			
No	2,215	1.46	1.43–1.50
Yes	456	1.34	1.27–1.43

Current smoking status			
Never	1,650	1.32	1.28–1.36
Former	505	1.58	1.50–1.66
Current	516	1.77	1.68–1.87

Alcohol consumption in preceding 12 months			
< 1 drink/week	1,981	1.41	1.37–1.45
≥ 1 drinks/week	690	1.54	1.47–1.62

Any supplement use in past month			
No	1,248	1.47	1.41–1.52
Yes	1,423	1.42	1.38–1.47

Calcium intake^b^			
< DRI	2,041	1.49	1.45–1.53
≥ DRI	564	1.28	1.22–1.34

Iron intake^b^			
< DRI	1,416	1.35	1.30–1.39
≥ DRI	1,141	1.58	1.53–1.64

Vitamin D supplement use in preceding month			
No	2,335	1.44	1.41–1.48
Yes	336	1.43	1.35–1.52

Exercise in preceding 30 days			
No exercise	1,197	1.65	1.59–1.71
Moderate	723	1.38	1.32–1.44
Vigorous	751	1.22	1.17–1.28

Muscle-strengthening activities in preceding 30 days			
No	2,093	1.51	1.46–1.55
Yes	578	1.24	1.18–1.30

Current hormone use			
No	2,058	1.52	1.47–1.56
Yes	613	1.22	1.17–1.28

Parity (live births)			
None	488	1.15	1.09–1.22
1	370	1.44	1.35–1.54
2	645	1.39	1.33–1.46
≥ 3	1,168	1.62	1.56–1.68

Surgical menopause			
No surgery	2,084	1.39	1.35–1.42
Hysterectomy, no bilateral oophorectomy	318	1.63	1.53–1.74
Oophorectomy	269	1.71	1.58–1.84

a Blood levels differed significantly (*p* < 0.01) across variable categories except for any supplement use (*p* = 0.50) and vitamin D supplement use (*p* = 0.92), Kruskal–Wallis test.

b The following variables had missing observations: calcium intake (*n* = 66) and iron intake (*n* = 114).

**Table 2:** Unadjusted and adjusted linear regression models for the association between tertiles of bone formation (serum BAP) and resorption (creatinine-adjusted urinary NTx) and percent difference in geometric mean BLLs, NHANES 1999–2002 (*n* = 2,671).

		Unadjusted model^a^		Final model^a^,^b^	
Tertile	*n*	Percent difference	95% CI	Percent difference	95% CI
NTx (nM bone collagen equivalent/mM creatinine)					
0–27.3	891	Reference		Reference	
27.40–40.76	890	4.96	−0.39 to 10.59	5.45	0.13 to 11.06
40.77–934.58	890	25.34	17.42 to 33.79	18.03	11.63 to 24.78

BAP (μg/L)					
0–10.30	901	Reference		Reference	
10.31–14.40	886	11.55	2.91 to 20.91	7.48	1.09 to 14.28
14.41–153.00	884	38.91	29.80 to 48.65	20.37	13.68 to 27.44

a NTx and BAP were modeled independently.

b Model adjusted for age, menopausal group, race/ethnicity, current hormone use, surgical menopause, smoking status, and BMI.

**Table 3:** Adjusted linear regression models for the association between tertiles of bone formation (serum BAP) and resorption (creatinine-adjusted urinary NTx) and percent difference in geometric mean BLLs stratified by menopausal status, NHANES 1999–2002.

	Premenopausal^a^ (*n* = 1,359)			Postmenopausal^b^ (*n* = 1,254)		
Tertile	*n*	Percent difference	95% CI	*n*	Percent difference	95% CI
NTx (nM bone collagen equivalent/mM creatinine)						
0–27.39	507	Reference		369	Reference	
27.40–40.76	480	0.64	−5.42 to 7.08	387	15.46	5.18 to 26.75
40.77–934.58	372	9.51	0.60 to 19.21	498	33.90	23.31 to 45.39

BAP (μg/L)						
0–10.30	569	Reference		319	Reference	
10.31–14.40	479	6.06	−1.52 to 14.22	384	12.87	1.12 to 25.99
14.41–153.00	311	14.02	6.49 to 22.08	551	30.03	17.93 to 43.36

a NTx and BAP modeled independently and adjusted for age, race/ethnicity, current hormone use, smoking status, and BMI.

b NTx and BAP modeled independently and adjusted for age, race/ethnicity, current hormone use, smoking status, BMI, surgical menopause, supplement use, and alcohol consumption.

**Table 4:** Adjusted linear regression models for the association between micronutrient intake or supplement use and percent difference in geometric mean BLLs among postmenopausal women, NHANES 1999–2002.

		Independent model^b^		Single model^c^	
Micronutrient/supplement^a^	*n*	Percent difference	95% CI	Percent difference	95% CI
NTx model					
Any supplement use	1,254	−11.81	−18.51 to −4.56	NA	
Calcium intake	1,226	−9.50	−19.58 to 1.84	−8.39	−18.21 to 2.60
Iron intake	1,216	−5.07	−12.63 to 3.15	−2.20	−9.38 to 5.53
Vitamin D supplement use	1,254	−12.52	−17.60 to −7.13	−12.52	−16.88 to −6.68

BAP model					
Any supplement use	1,254	−12.84	−19.75 to −5.33	NA	
Calcium intake	1,226	−9.72	−19.64 to 1.43	−8.66	−18.28 to 2.11
Iron intake	1,216	−4.76	−11.85 to 2.90	−1.77	−8.46 to 5.40
Vitamin D supplement use	1,254	−12.77	−17.87 to −7.35	−12.15	−17.15 to −6.85

NA, not applicable.

a Calcium and iron intake were dichotomized at the age-specific DRI level (≥ DRI vs. < DRI). Any supplement use and vitamin D supplement use were dichotomized (yes vs. no).

b Independent models for percent difference in geometric mean BLLs for each micronutrient/supplement, adjusted for NTx (NTx models) or BAP (BAP models), age, race/ethnicity, current hormone use, smoking status, BMI, surgical menopause, and alcohol consumption.

c Single models (*n* = 1,210) for percent difference in geometric mean BLLs for all micronutrients, including calcium, iron, and vitamin D, and adjusted for NTx (NTx model) or BAP (BAP model), age, race/ethnicity, current hormone use, smoking status, BMI, surgical menopause, and alcohol consumption.
